# Dictation and vocabulary knowledge tests for adult native Chinese readers

**DOI:** 10.3758/s13428-025-02669-4

**Published:** 2025-04-22

**Authors:** Yiu-Kei Tsang

**Affiliations:** Department of Education and Psychology, Academy of Wellness and Human Development, Hong Kong Baptist University, Kowloon Tong Kowloon, Hong Kong

**Keywords:** Language proficiency, Individual differences, Dictation test, Vocabulary test, Chinese language

## Abstract

To examine how individual differences in language skills affect language processing, it is essential to have good-quality tests that can assess such individual differences accurately. This study introduces a dictation test and a vocabulary knowledge test in Chinese, which aim to measure lexical expertise in proficient Chinese language users like university students. The psychometric properties of the two tests were examined with two groups of participants. In the first group, exploratory factor analyses confirmed that each of these tests was unidimensional, measuring a single underlying construct of lexical expertise. After removing some problematic items, the two tests also demonstrated satisfactory internal reliabilities. Although the test scores were only weakly correlated with self-reported measures of language proficiency, the correlation with word recognition performance was moderate. These results were successfully replicated with the second cross-validation group, confirming the reliability and convergent validity of the tests. An additional dataset further showed that the vocabulary test score was positively correlated with sentence comprehension performance. Taken together, the tests have acceptable psychometric quality and can serve as tools for examining individual differences in Chinese language processing. The tests are freely available online, and normative performance data are provided, facilitating their use in future research.

**Supplementary Information:**

The online version contains supplementary material available at 10.3758/s13428-025-02669-4.

## Introduction

Following the tradition of experimental psychology, psycholinguistic studies typically involve manipulating certain linguistic variables. Items in different experimental conditions are presented to participants, and conclusions are drawn based on differences in the averaged responses across these conditions. The resulting theories are considered to describe mechanisms of language processing that are applicable to everyone in the target population. However, it is well known that few experimental manipulations have the same effect on all individuals. Instead, these effects often vary greatly in strength and, in some cases, can manifest in opposite directions, even within seemingly homogeneous groups like university students (Miller & Schwarz, 2018). These individual differences are often considered random noise in data analyses and are of little theoretical interest (Speelman & McGann, 2013). In psycholinguistics, although there has been early evidence of systematic individual differences in various aspects of language use (e.g., Dąbrowska & Street, 2006; Dixon et al., 1988; Kuperman & Van Dyke, 2011), the investigation of individual differences has remained mostly a concern in fields like language development and second language (L2) education (Binder et al., 2017). For example, in a recent meta-analysis of individual differences in vocabulary knowledge and reading comprehension in Chinese (Dong et al., 2020), all 81 studies included concerned developing readers in primary and secondary schools or adult L2 Chinese learners. None has tested proficient native adult Chinese users. This creates a gap in the understanding of how individual variability shapes language use.

Language processing depends not just on linguistic factors but also on individual characteristics, such as working memory and vocabulary knowledge (Dixon et al., 1988). Acknowledging the limitations of the traditional experimental approach, there has been a growing interest within psycholinguistics in studying individual differences. This shift is supported by three trends in the field. Firstly, linear mixed-effects (LME) modeling has become the standard approach in data analysis (Baayen et al., 2008), enabling researchers to explicitly test for variability in the size of fixed effects across participants (i.e., the by-participant random slopes). After all, attempts to examine whether individual differences systematically modulate language processing will only be meaningful when there are reliable individual differences in the first place (Staub, 2021). Secondly, the availability of large-scale datasets provides the statistical power needed to model complex interactions between individual characteristics and linguistic factors. For instance, using data from the English Lexicon Project (ELP; Balota et al., 2007), Yap et al. (2012) found that participants with better vocabulary knowledge were less sensitive to neighborhood size. In a similar vein, Lim et al. (2020) reanalyzed data from the Chinese Lexicon Project (CLP; Tse et al., 2017) and observed stronger effects of word frequency and semantic transparency among participants with higher lexical fluency. Finally, in the spirit of open science, researchers are increasingly willing to make their tests of language abilities openly accessible. This development is particularly beneficial to psycholinguists who study proficient adult language users, where the number of available tests has been limited. Sometimes, researchers have to rely on tests originally designed for developing or L2 learners (e.g., LexTALE; Lemhöfer & Broersma, 2012), which may result in ceiling performance in native adult users. For example, the native English participants scored over 90% correct in the LexTALE test in an eye-tracking megastudy developed by Cop et al. (2017).

Andrews et al. (2020) presented an example of making their tests available to the research community. Specifically, they described the spelling dictation and spelling recognition tests that the research group has used to explore how native speakers’ lexical expertise influences various aspects of language processing, including overall reading proficiency (Andrews, 2008) and sensitivity to orthographic, morphological, and semantic information (Andrews & Hersch, 2010; Andrews & Lo, 2013; Andrews et al., 2017). Building upon these prior works, Eskenazi et al. (2023) further refined the items used in the dictation test and came up with 20 items with low measurement error and good discriminative power. Similarly, Vermeiren et al. (2023) provided a comprehensive “walkthrough” detailing the development, refinement, and validation of several English language tests (e.g., vocabulary knowledge and reading comprehension) specifically developed for university students. Tests for more complex language use, such as syntactic processing, have also been developed recently (Chernova et al., 2023; Dąbrowska, 2018), although the psychometric properties (e.g., factor structure and internal reliability) of these tests have not been thoroughly evaluated. These resources save researchers valuable time and effort that would otherwise be spent developing their own assessments, thereby improving the efficiency of testing individual differences in language processing.

Inspired by these previous works, this report aims to introduce and make available two tests of lexical expertise for proficient adult readers of simplified and traditional Chinese. The first test is a dictation task, which requires participants to accurately produce the written forms of some Chinese words they hear. In contrast to a word recognition task, dictation may more directly tap into the precision of one’s orthographic representations because partial orthographic knowledge cannot produce the correct answers (Andrews et al., 2020; Yates & Slattery, 2019). The second test is a vocabulary knowledge test, which reflects one’s ability to recognize the meanings (definitions) of low-frequency Chinese words without the support of sentential contexts. While there are different ways to measure vocabulary knowledge, it is widely recognized as a key factor to reading performance in both developing and proficient readers (Binder et al., 2017; Dong et al., 2020; Zhang & Zhang, 2022).

The dictation and vocabulary knowledge tests were originally designed as an assessment tool of Chinese language proficiency in the MEgastudy of Lexical Decision (MELD) project, which aims to provide large-scale datasets of Chinese word recognition. The first output of the project, MELD-SCH (Tsang et al., 2018), includes data of 12,587 words collected from proficient adult readers (i.e., university students) of simplified Chinese. Unlike CLP, which focuses on either one-character or two-character words (Sze et al., 2014; Tse et al., 2017, 2023), MELD-SCH contains Chinese words of one-character to four-character long, enabling researchers to examine the less explored topic of word length in Chinese. Along the same line, data was also collected from university students in Hong Kong, who were presented with the same set of items in traditional Chinese (Tsang et al., 2024). As identical items were tested in the two scripts, using the same instruction and experimental setup, the dataset can support a direct comparison between the processing of simplified and traditional Chinese. A smaller-scale dataset E-MELD, which includes the event-related potential (ERP) recording of 1020 two-character words in traditional Chinese, was also developed to support a more fine-grained analysis of the temporal locus of different effects (Tsang & Zou, 2022).

Just as the participants in ELP were administered a vocabulary knowledge test to enable further analyses of individual differences (Balota et al., 2007; Yap et al., 2012), we envisioned to examine individual differences in Chinese word recognition in the MELD project. Although a LexTALE-style test has recently been developed in Chinese (LexCHI; Wen et al., 2024), we were unaware of the existence of any freely available tests when the MELD data was collected in 2014–2016. At that time, researchers had to either purchase expensive assessment tools, such as the Verbal Comprehension scale from the WAIS-IV, use tests developed for L2 learners (e.g., Sze et al., 2014), or creatively repurpose available data to provide an index of individual differences. For instance, Lim et al. (2020) combined participants’ speed and accuracy in the lexical decision task to create a measure of “lexical fluency”. For the MELD project, we chose the alternative approach of developing our own tests. In designing the dictation and vocabulary tests, we had three main goals. Firstly, the tests needed to be challenging enough to distinguish between university students, who are the major source of participants in psycholinguistic research. Secondly, they could be used with different groups of Chinese speakers, including those from various geographical regions and those using different scripts (i.e., simplified vs. traditional Chinese). Thirdly, the tests needed to be short, ideally taking no more than 10 min, to ensure they could be easily integrated into a wide range of studies.

In Tsang et al. (2024), we reported that after controlling for major lexical factors (e.g., word frequency and word length), performance on the dictation test predicted accuracy rates in the lexical decision task, although the effect on response times was non-significant. This finding supports the utility of the dictation test. However, we did not examine the effects of the vocabulary knowledge test in that study nor make the tests available to other researchers. More importantly, the psychometric properties of these tests had not been adequately evaluated prior to conducting the analyses. Given that establishing good psychometric properties, such as reliability and validity, are crucial before the tests can be used and interpreted meaningfully, we provide relevant information in this report, following the recommended steps outlined in Brysbaert’s (2024) tutorial.

## Method

### Participants

Data were collected from 930 native Chinese who took part in the MELD project (Tsang et al., 2018, 2024). All participants were university students, but their language backgrounds were somewhat diverse. Approximately 54% of them (*N* = 504) were from three universities in Guangzhou, China: South China Normal University, South China Agricultural University, and South China University of Technology. These participants came from various regions of China and could speak their local dialects (e.g., Cantonese for those from Guangdong and Shanghainese for those from Shanghai). However, Mandarin is the medium of instruction (MOI) in their formal education. Furthermore, although English has been a popular foreign language subject in mainland China’s curriculum, most of these participants had limited exposure to English outside of school. Participants were recruited through mass e-mail and word-of-mouth at South China Normal University. They were provided a small monetary compensation (RMB 20/h) for participation.

The remaining 46% (*N* = 426) of the participants were from The Chinese University of Hong Kong, where English is the MOI. All of them were native Cantonese speakers born and raised in Hong Kong. In pre-tertiary education, most schools in Hong Kong used Cantonese as the MOI, although some higher-ranking schools used English. Cantonese is the primary spoken language in daily life, but written English is common in formal settings (e.g., business documents or road signs) because it is an official language in Hong Kong. Additionally, Hong Kong’s language education policy requires students to learn Mandarin in primary and secondary schools, so all participants could speak Mandarin, though their proficiency levels varied. Entertainment from mainland China and Taiwan, such as TV shows and pop songs, was also popular in Hong Kong, providing informal exposure to Mandarin. Participants were recruited through mass e-mail and word-of-mouth at The Chinese University of Hong Kong. They were provided a small monetary compensation (HKD 50/h) for participation.

The goal of this paper is to report the psychometric properties of the vocabulary and dictation tests developed in the MELD project. To this end, the 930 participants were divided into two groups for analysis. The first group, comprising 400 randomly chosen participants, as recommended by Brysbaert, 2024, provided initial data for evaluating the psychometric properties of the vocabulary and dictation tests. The second group, consisting of the remaining 530 participants, served as an independent group for cross-validation and provided a distribution norm for test performance.

## Materials

Participants from mainland China and Hong Kong used simplified and traditional Chinese, respectively. Their spoken Chinese also took different forms (Mandarin vs. Cantonese), but their written Chinese conformed to the modern standard known as “Baihua” (白話). There was also frequent cultural exchange between mainland China and Hong Kong, especially after the handover in 1997. Consequently, vocabulary and grammatical rules were largely consistent across the written Chinese of all participants. Indeed, Tse et al. (2017) showed that the frequency count of SUBTLEX-CH (Cai & Brysbaert), which was originally developed for simplified Chinese, could explain a large proportion of variance in the lexical decision performance in CLP, whose participants were traditional Chinese users in Hong Kong. The commonality set the foundation to develop tests of Chinese proficiency that are applicable to different groups (e.g., geographical locations, dialects, and scripts) of Chinese users.

Materials for the tests were selected through several steps. The final set of items used in the dictation and vocabulary tests can be found in the Appendix. To make the tests more readily available to interested researchers, two Microsoft Word documents (.docx), one in simplified Chinese and the other in traditional Chinese, which show the instruction and the presentation format of the tests together with the four choices for the vocabulary test, are freely downloadable here: https://osf.io/v26nk/.

### **Dictation test**

The dictation test is designed to assess participants’ ability to produce written Chinese words accurately. Participants are required to write down each of the words an experimenter produces orally. The test was developed with the assistance of two Chinese language teachers. The first teacher, a native Cantonese speaker, had 2 years of experience teaching in a Hong Kong secondary school and 2 years teaching at a university. The second teacher, a native Mandarin speaker from mainland China, was employed as a Mandarin teacher at a Hong Kong university with 5 years of teaching experience. They were asked to work together and selected 10 to 20 words from SUBTLEX-CH (Cai & Brysbaert, 2010) for the dictation test. Minimal instructions were given when they chose the words. The only criteria included, firstly, the words chosen must be challenging enough to avoid ceiling-level performance among university students, who generally have high language proficiency. Therefore, the teachers focused on low-frequency words rather than selecting words from various frequency bands, as was done in prior studies (e.g., Wen et al., 2024). Secondly, only multi-character words were chosen to avoid ambiguity due to homophonic characters, which are common in Chinese. Thirdly, although the written vocabulary is mostly shared between Hong Kong and mainland China, some regional variations do exist. For example, “butter” is “黃油” (literally “yellow-oil”) in mainland China and “牛油” (literally “cow-oil”) in Hong Kong. The two teachers were advised to avoid such words to ensure that the test can be used in different regions. In a post hoc discussion with the first teacher, he mentioned that he picked several words that his students often got confused based on his experiences of teaching (e.g., item d02 “安詳” was frequently miswritten as “安祥”).

In the end, the two teachers came up with a pool of 15 words, consisting of nine two-character words, two three-character words, and four four-character words (total number of characters = 40). Scoring of the dictation test is character-based. Participants get one point for each correctly written character. As shown in the Appendix, although all items are of low frequency, some constituent characters are simple and of high frequency. The original intention for including these easy characters was to ensure that participants would not be too frustrated when completing the test. The author of this article, who identified himself as having an above-average Chinese proficiency, completed the test himself and scored 32 out of 40 (i.e., 80% correct). The test was thus believed to be sufficiently difficult for university students.

### **Vocabulary test**

The vocabulary test takes a multiple-choice format. It is designed to assess participants’ ability to recognize the meanings of written Chinese words without contextual support. In each item, a target word and four alternative meanings are provided. Participants are required to pick the choice that best describes the word’s meaning. The author selected 60 low-frequency target words from SUBTLEX-CH (Cai & Brysbaert, 2010). For the four alternative choices, the correct answer was developed based on the definition of Baidu Dictionary. In some cases, a slight modification was made to simplify or improve the clarity of the definition. For example, the definition of the item v8 “種羊” provided by the dictionary is a complete sentence “種羊即留作種用的羊” (Breeding sheep is sheep reserved for breeding). It was modified into “用作配種的羊” (sheep for breeding). The distractor choices were then constructed by the author. This initial set of items was screened by the two teachers who helped construct the dictation test. Based on their advice, 14 items were discarded because of inappropriate difficulty levels, and the choices for 12 items were modified to improve clarity (i.e., making the alternative choices look less similar). Fifteen university students in Hong Kong were invited to complete the remaining 46 items. The purpose of the vocabulary test was clearly explained to them, and they were encouraged to provide qualitative feedback on the items. Based on the results of this pilot testing, items that were too easy or difficult (more than 80% or less than 20% correct)^1^ were removed. In addition, an item was also discarded if it received five or more negative comments (e.g., “the word was too technical”, or “the alternative choices were confusing”) from the pilot participants. In the end, 24 items were retained. The scoring of this test is item-based. Participants get one point for each correctly answered item.

### **Language background survey**

A survey was designed to inquire about participants’ demographic information, including age, gender, the university they were studying at, and their major subject. The survey also asked participants to report the estimated number of hours of Chinese reading per week (excluding academic reading), their self-perceived Chinese proficiency (from 1 to 10, higher score = higher proficiency level), the number of foreign languages they know, and their Chinese subject score in public examination.

Elaboration is needed regarding the Chinese subject score in public examinations. Although secondary school students in both mainland China and Hong Kong need to complete a centralized public examination for university entrance, the curriculum of the Chinese subject and the format of the examination differ. Specifically, China students complete the Nationwide Unified Examination for Admissions to General Universities and Colleges (commonly referred to as “Gaokao”, literally meaning “Higher Examination”), while Hong Kong students complete the Hong Kong Diploma of Secondary Education Examination (“HKDSE”). The Chinese language is a core subject in both examinations. At the time of data collection (2014–2016), Gaokao emphasized reading comprehension and composition, while HKDSE also tested listening comprehension and oral skills. The scoring system also differs. Gaokao directly scores a student on a continuous scale (maximum score = 150). In contrast, HKDSE only provides an ordinal performance banding, which, in ascending order of proficiency level, are 1, 2, 3, 4, 5, 5*, and 5** (in the present study, 5* and 5** were recoded as 6 and 7, respectively). Despite these differences, the public examinations were considered to indicate the overall level of Chinese language proficiency within each region.

## Procedure

In the MELD project, each participant completed four tasks in their respective scripts. These tasks included a dictation test, a vocabulary test, a language background survey, and a lexical decision task. Details of the lexical decision task have been reported in Tsang et al., (2018, 2024). Data collection took place in computer laboratories at South China Normal University for participants from mainland China and at The Chinese University of Hong Kong for participants from Hong Kong. Before the study, all participants were provided with written information about the procedure and consented to participate voluntarily. Then, participants completed the dictation test. At each testing site, a trained experimenter was responsible for orally presenting the words. The experimenter at South China Normal University read the words in Mandarin, while the one at The Chinese University of Hong Kong read them in Cantonese, ensuring that the language used was appropriate for the respective participant groups. The experimenter read aloud each word twice. Participants were given 15 s (for two-character words) or 30 s (for three-character and four-character words) to write down the words. After the dictation test, the participants completed the vocabulary test, the language background survey, and the lexical decision task in order and in a self-paced manner.

## Analyses and results

### Group 1: Initial evaluation of psychometric properties

**Fig. 1:**
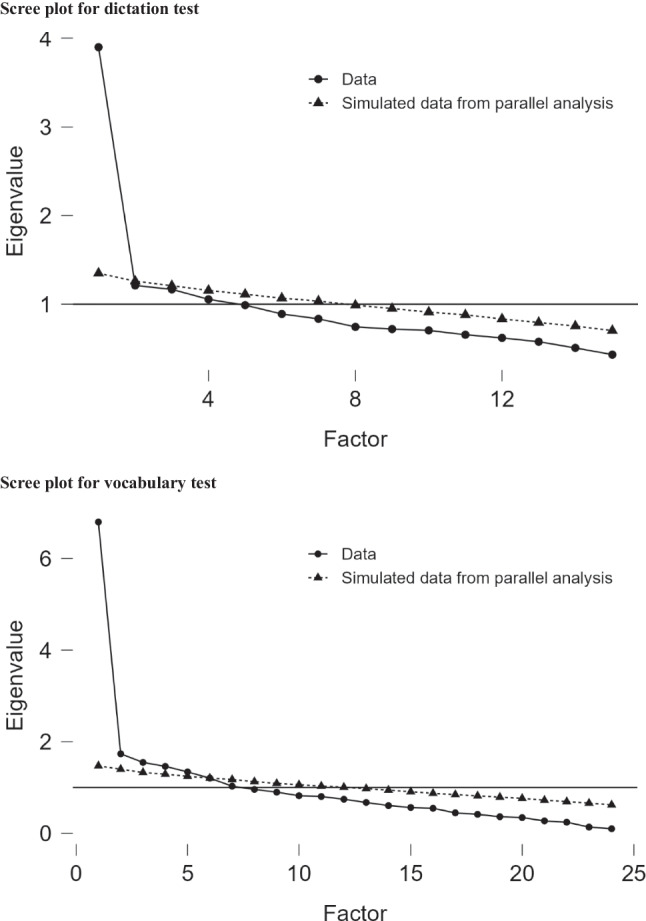
Scree plots of the dictation test (*top*) and vocabulary test (*bottom*)

Analyses of the psychometric properties of the dictation and vocabulary tests were done using JASP 0.17.2.1 (JASP Team, 2024). First, the dimensionality of the dictation test was evaluated using exploratory factor analysis (EFA). The test scoring was based on the number of characters correctly written,^2^ which was affected by word length and could not be directly analyzed. Therefore, the score of each item by each participant was converted into proportion correct within a word. For example, if a participant wrote down both characters of a two-character word correctly, he/she scored 1 (2/2) for this item. Similarly, if he/she got two characters correct in a three-character word, the score would be 0.67 (2/3). The converted data was used in the EFA. The scree plot (Fig. 1, top) shows that although several factors have an eigenvalue above 1, only the first factor has an eigenvalue much higher than the simulated parallel analysis. There is also a large drop in explanatory power from the first to the second factor. Based on these observations, the dictation test is considered to be unidimensional (i.e., it reflects one latent construct). Table 1 lists the factor loadings of the 15 items of the test.

**Table 1:** Factor loadings and item–rest correlation of items in dictation test in Group 1

Item_id	Factor loading	Item–rest correlation
d06	0.741	0.616
d05	0.605	0.512
d13	0.544	0.479
d08	0.523	0.450
d12	0.496	0.414
d07	0.488	0.398
d14	0.480	0.421
d04	0.422	0.377
d02	0.408	0.380
d03	0.405	0.345
d10	0.396	0.352
d15	0.382	0.282
d09	0.376	0.311
d01	0.140	0.115
d11*	0.100	0.080

The item marked with an asterisk (*) showed low factor loading and item–rest correlation. It was removed from the reliability analysis

Second, the internal reliability of the dictation test was evaluated based on the single-factor structure model. Table 1 also shows the item–rest correlation of the 15 items. Items with low factor loadings (< 0.1) and/or low item–rest correlation (< 0.1) are problematic and would be discarded. The cutoff at 0.1 is admittedly lenient, and was decided to keep more items (i.e., only very bad items would be removed) because the total number of items was initially small. Among the 15 items, item d11 has very low factor loading (0.10) and item–rest correlation (0.08), indicating that it might not be a usable item. Removing d11 resulted in better reliability values. Specifically, the Cronbach’s α and McDonald’s ω of the test equaled 0.761 and 0.771, respectively, indicating a satisfactory level of reliability. It is also noteworthy that removing further items would not greatly improve the reliability of the test (the best improvement is 0.01; see the online output at https://osf.io/v26nk/).

Similar analyses were done with the vocabulary test. Because each item in the test was scored as either correct (1) or incorrect (0), the EFA was performed with a tetrachoric correlation matrix. The scree plot (Fig. 1, bottom) shows that five factors have eigenvalues above 1, which are also higher than the simulated parallel analysis. However, the second through the fifth factors have much lower eigenvalues than the first factor. In addition, Table 2 shows that several items (v01, v06, v07, and v10) have negative or low factor loadings (< 0.1) and low item–rest reliability (< 0.1). Removing these items turns the scree plot more supportive of a one-factor model (see the online output at https://osf.io/v26nk/). Therefore, it is believed that the remaining items reflect a single latent construct.

**Table 2:** Factor loadings and item–rest correlation of items in vocabulary test in Group 1

Item_id	Factor loading	Item–rest correlation
v02	0.870	0.557
v05	0.848	0.569
v12	0.775	0.540
v15	0.726	0.504
v13	0.624	0.411
v21	0.602	0.365
v11	0.598	0.388
v19	0.570	0.374
v14	0.559	0.396
v24	0.548	0.360
v03	0.524	0.289
v16	0.522	0.382
v09	0.508	0.337
v08	0.464	0.321
v22	0.373	0.283
v10*	– 0.309	– 0.166
v17	0.296	0.205
v23	0.273	0.205
v20	0.250	0.179
v04	0.226	0.186
v18	0.194	0.132
v01*	0.065	0.083
v07*	0.064	0.077
v06*	– 0.022	0.044

The items marked with an asterisk (*) showed low or negative factor loadings and item–rest correlations. They were removed from the reliability analysis

The reliability of the vocabulary test was estimated based on the single-factor model. After removing the problematic items (v01, v06, v07, and v10), the Cronbach’s α and McDonald’s ω of the test were 0.798 and 0.802, respectively, indicating good internal reliability. As in the case for the dictation test, removing further items would not greatly improve the reliability of the vocabulary test (the best improvement is 0.005; see the online output at https://osf.io/v26nk/).

Table 3 presents the correlations between the two tests (test scores calculated after removing the problematic items) and other language-related measures. Most correlations were modest in size but statistically significant and in the expected direction. Better performances on both the dictation and vocabulary tests were associated with more Chinese reading per week, higher self-rated Chinese proficiency, and faster, more accurate lexical decision responses. Given that participants from mainland China and Hong Kong took different public examinations (Gaokao vs. HKDSE), correlations between the test scores and Chinese examination scores were calculated separately. For participants from China, the correlations were non-significant. However, for the Hong Kong group, there were moderate and positive correlations (Spearman’s rho = 0.36 and 0.47). It is also important to note that there were 12 lists for the lexical decision task, and each participant completed only one, which could potentially introduce noise into the correlation analyses. Yet, the strongest correlations with the dictation and vocabulary tests were found with error rates in the lexical decision task (both Pearson’s *r*s = – 0.54). Finally, the two tests were themselves moderately correlated (Pearson’s *r* = 0.63, *p* < 0.001).

**Table 3.**
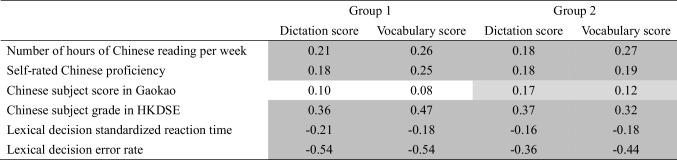
Correlations between the dictation and vocabulary tests and other language-related measures

Significant correlations are shaded (dark grey: *p* <.001; light grey: *p* <.05). The values are Pearson’s *r*, except the correlation with Chinese subject grade in HKDSE, which is Spearman’s rho. The Chinese subject score in Gaokao and Chinese subject grade in HKDSE are used to represent the performance in public examination of mainland China and Hong Kong participants, respectively. The lexical decision reaction time used in the analysis is standardized (*z*-score transformed) within each participant to eliminate individual differences in overall processing speed

The initial evaluation of the psychometric properties of the dictation and vocabulary tests indicates that, after removing some problematic items, the tests have satisfactory reliability. Participants who excelled in these tests generally also had better performance in other measures of Chinese proficiency. Although most correlations were modest, the correlation with the error rate in lexical decision was stronger (around 0.5), which was comparable to the strength of correlations observed in previous reports of language tests for proficient adult readers (e.g., Andrews et al., 2020; Vermeiren et al., 2023). Therefore, it is believed that the two new tests are potentially useful as a measurement of Chinese proficiency, particularly in contexts where word recognition accuracy is of interest.

### Group 2: Cross-validation with an independent group

The initial evaluation of psychometric properties using Group 1 participants (*N* = 400) was cross-validated with Group 2 participants (*N* = 530). First, confirmatory factor analyses (CFA) were conducted to confirm the single-factor structure of both tests. Following Brysbaert (2024), four fit measures are reported (for other indices, see the online output at https://osf.io/v26nk/), namely CFI, TLI, RMSEA and SRMR. CFI and TLI indicate goodness-of-fit and values above 0.90 and 0.95 are considered acceptable and good, respectively. RMSEA and SRMR indicate error and values below 0.08 and 0.05 are considered acceptable and good.

For the dictation test, after removing the problematic item (d11), the single-factor model provides an acceptable or good fit to the data (CFI = 0.944, TLI = 0.934, RMSEA = 0.044, and SRMR = 0.041). The reliability indices also indicate satisfactory reliability (Cronbach’s α = 0.790; McDonald’s ω = 0.786). Similarly, for the vocabulary test, after removing the problematic items (v01, v06, v07, and v10), the single factor model provides an acceptable or good fit to the data (CFI = 0.982, TLI = 0.980, RMSEA = 0.024, and SRMR = 0.062). The reliability indices indicate satisfactory reliability (Cronbach’s α = 0.801; McDonald’s ω = 0.780). Together, the unidimensional nature and satisfactory internal reliability of the dictation and vocabulary tests in the initial analyses could be replicated with an independent group of participants.

Table 3 also shows the correlation between the dictation and vocabulary tests and other proficiency measures for the cross-validation Group 2. The Pearson’s *r* between the two tests was 0.63, which is identical to the value of the initial analysis with Group 1. All other correlations were modest in size but statistically significant, including the previously non-significant correlations with Gaokao score. On the other hand, the correlations with error rate in lexical decision were somewhat smaller, indicating a potential of over-estimation in the Group 1 analysis. Interestingly, in developing the LexCHI test, Wen et al. (2024) also observed a similar shrinkage in correlation in their cross-validation group (see Tables 3 and 4 vs. Table 8 in Wen et al., 2024). Yet, considering that all correlations were in the same direction and of mostly similar sizes between the two groups, it is believed that the cross-validation in an independent group was overall successful.

**Table 4.**
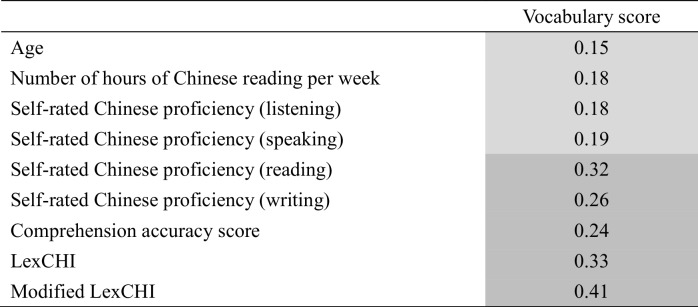
Correlations between vocabulary score and other language performance measutres in the additional eye-tracking datase

Significant correlations are shaded (dark grey: *p* <.001; light grey: *p* <.05). The values presented are Pearson’s *r*

**Fig. 2:**
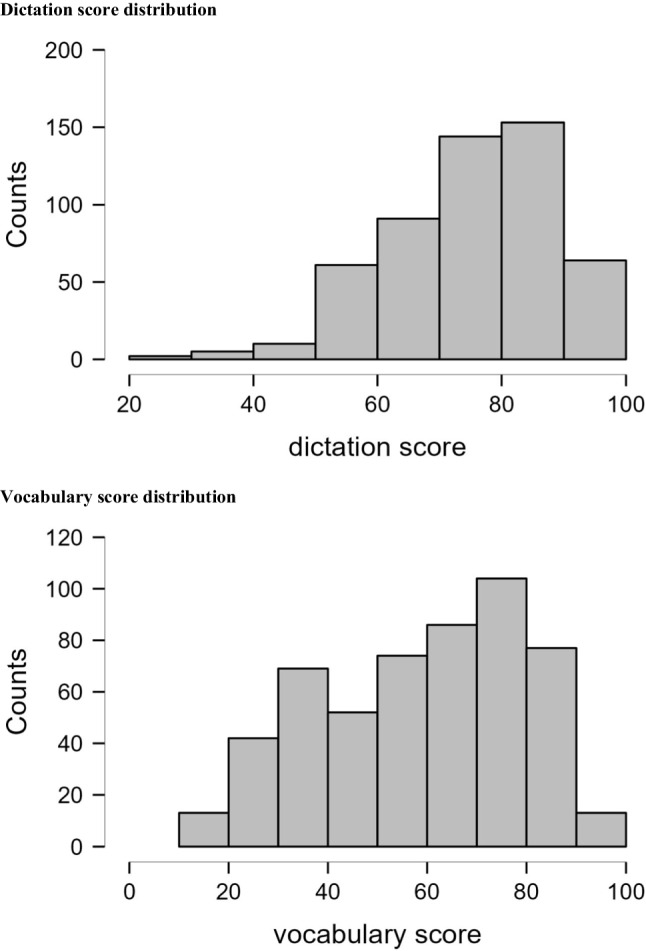
Distribution of dictation test score (*top*) and vocabulary test score (*bottom*)

Figure 2 illustrates the distribution of the dictation and vocabulary test scores (on a % correct scale) for the Group 2 participants. The distributions were obtained from over 500 participants and can serve as performance norms for the tests. The effort in creating sufficiently difficult tests was partially successful, as evidenced by a broad range of participants’ scores. In particular, the 75 th percentiles of the dictation and vocabulary scores are 85.71 and 80, respectively, which are far from ceiling. However, the distributions are still somewhat skewed towards the lower end, particularly for the dictation test (skewness = – 0.71 and – 0.34 for the dictation and vocabulary scores, respectively). This may be attributed to the fact that some of the low-frequency words used contain simple and high-frequency characters (e.g., “一” in “一蹶不振”). While further development of the tests can include items with more difficult characters to reduce the skewness, it should be noted that the psychometric properties of the dictation test were mostly satisfactory even with these easy characters.

### Further analyses with the vocabulary test

An additional dataset is available to demonstrate further the utility of the vocabulary test in measuring lexical expertise. In a series of eye-tracking experiments of Chinese sentence reading, participants were asked to complete the present 20-item vocabulary test (i.e., problematic items have been discarded), LexCHI (Wen et al., 2024), a modified LexCHI (Tsang et al., preprint),^3^ and rated their Chinese proficiency level in speaking, listening, reading and writing. After that, they read 130 to 180 Chinese sentences as their eye movements were recorded. A true-or-false comprehension question was given after ~ 25% of the sentences to ensure attentive reading, which enables the calculation of a sentence comprehension score (i.e., the percentage correct in answering the comprehension questions). For the present purpose, the vocabulary score was correlated with other language performance measures. It is noteworthy that there are several differences between this additional dataset with the afore-reported one based on MELD. Firstly, the participants were recruited from the community. They had a diverse range of education levels and were either young (age below 30) or old (age above 60) readers. This helps test whether the vocabulary test can be extended beyond the university student sample. Secondly, a comprehension test and the previously validated LexCHI test was used, providing additional benchmark other than lexical decision performance. Thirdly, instead of asking participants to rate their overall Chinese proficiency level, they were asked to rate specific language skills. It is expected that, as the vocabulary test is a written one, its score may correlate better with visual domain skills (reading and writing) than auditory ones (listening and speaking).

The sample size of the additional dataset is 188 (female = 114; male = 74; mean age = 48.56). The majority of the participants had an education level of secondary school or below (117), while 42 and 29 had completed university and postgraduate studies, respectively. The mean performance in the vocabulary test is 50.93 (SD = 18.73). Unsurprisingly, participants of different education levels also differed in their mean vocabulary scores (*F*(2,185) = 7.93, *p* < 0.001). Post-hoc comparison with Tukey correction showed that participants who had attained a postgraduate level of education obtained higher scores than those with an education level of secondary or below (61.38 vs. 47.22, *p* < 0.001), but the comparisons involving university-level participants (54.05) failed to reach statistical significance.

Table 4 displays the correlation between the vocabulary test score and the other measures. There was a weak but significant positive correlation between vocabulary score and age (Pearson’s *r* = 0.147, *p* < 0.05), which is consistent with the well-established increase in vocabulary knowledge as one accumulates language experiences with age (e.g., Keuleers et al., 2015). Moreover, although the correlation strengths were modest, the vocabulary score was positively related to all other measures. As expected, the correlations with reading and writing were numerically larger than those with listening and speaking. The correlation with comprehension score is particularly encouraging, as it indicates that the vocabulary score is related not only to word recognition (as reflected by lexical decision in the MELD-CH dataset) but also to sentence-level understanding. Finally, two factors were extracted in an EFA of the seven lexical expertise measures (Table 5). The self-reported measures came together as the first factor. More importantly, the present vocabulary test was grouped with LexCHI and the modified LexCHI as the second factor, suggesting that they all measured the same underlying construct of lexical knowledge. Together, the additional dataset provides further evidence that the present vocabulary test can be used to measure Chinese lexical proficiency in a community sample.

**Table 5:** Factor loadings of the seven proficiency measures in the additional eye-tracking dataset

Test	Factor 1	Factor 2
Self-rated Chinese proficiency (listening)	0.885	
Self-rated Chinese proficiency (speaking)	0.874	
Self-rated Chinese proficiency (reading)	0.854	
Self-rated Chinese proficiency (writing)	0.759	
Modified LexCHI		0.786
LexCHI		0.696
Vocabulary test		0.494

Only factor loadings > 0.2 are shown to improve clarity. Details of the analyses and results are available at https://osf.io/v26nk/

## Discussion

Language processing depends on both linguistic factors and personal characteristics. To support the increasing interest in examining how individual differences in language skills modulate the sensitivity to linguistic variables among proficient native language users, researchers have worked to develop tests for different language abilities that are reliable and valid in proficient adult samples (e.g., Andrews et al., 2020; Vermeiren et al., 2023). Along this line, we reported the development of a dictation test and a vocabulary knowledge test for proficient Chinese users in this study. Both tests are short and can be completed in 10–15 min, facilitating the inclusion of these tests to measure lexical expertise in standard psycholinguistic experiments. Despite the relatively small number of items, both tests demonstrate satisfactory reliabilities after removing some problematic items. In addition, both tests are unidimensional, providing some support to their construct validity (i.e., measuring a single construct of lexical expertise). Finally, the tests also have reasonable convergent validity, as evidenced by the moderate and negative correlations between the test scores and the error rates in word recognition, and for the vocabulary test, positive correlations with comprehension scores and LexCHI scores.

The test scores are only weakly correlated with the overall self-rated Chinese proficiency level, although the correlations between the vocabulary score and self-rated proficiency are numerically stronger, especially in the visual domain (see Tables 3 and 4). The self-reported measures also have a relatively low correlation with the lexical decision and reading comprehension performance (*p*s < 0.17; see the outputs at https://osf.io/v26nk/). Consistent with this observation, Lim et al. (2020) also found self-reported proficiency to be a poor predictor to Chinese word recognition performance, which they attributed to range restriction in university participants. Alternatively, self-reported indices may simply be inaccurate indices of language proficiency. Although self-assessment is a popular method for measuring language proficiency (Olson, 2024), it is widely acknowledged that it can be biased by factors unrelated to real performance, such as anxiety, demand characteristics, different interpretations of the scale, or differences in the comparison group (e.g., MacIntyre et al., 1997; Neveu & Gollan, 2024; Tomoschuk et al., 2019). The self-reported measures were used for the MELD project because we could not find other readily available tests at the time of data collection. Again, this highlights the values of developing objective, performance-based language proficiency tests and making them available to the research community. In the case that self-reported measures are needed, it is advisable that the self-assessment should be made for each specific language skills, as illustrated by the results with the additional dataset.

Both the dictation and vocabulary knowledge tests aim to measure lexical expertise; that is, they reflect the ability to process isolated Chinese words. Accordingly, the two tests are themselves well correlated (Pearson’s *r* = 0.63), consistent with previous studies that examine the correlation between vocabulary knowledge and dictation performance (e.g., Andrews & Veldre, 2021; Eskenazi et al., 2023). On the other hand, there are also subtle differences in the skills measured by these tests. For instance, the dictation test may be most relevant to the precision of a word’s orthographic representation because incomplete knowledge of the written word forms will result in errors in dictation (Andrews et al., 2020). Particularly for the Chinese script, because of its logographic nature and the prevalence of homophonic characters, it is impossible to infer the correct orthographic form of a word based on its pronunciation. Indeed, visual-orthographic skill is important in Chinese reading development (McBride, 2016), while phonological skills like phonological awareness have a more limited role to play (Ruan et al., 2018). The present correlation between the dictation test scores and word recognition performance in adult Chinese users suggests that the relationship is not limited to young readers but also extends to proficient readers. Future studies will be meaningful in examining how variation in the dictation scores is related to other aspects of Chinese language processing.

The vocabulary test takes a multiple-choice format similar to the Nelson–Denny vocabulary test, which is a popular instrument in assessing individual differences of adult English users. The test requires participants to identify the correct meanings of isolated words and is thus related to semantic retrieval. Being able to identify the meanings of more words can be considered as being less likely to encounter novel words in reading. As such, the reader can directly retrieve the words’ meanings from memory, instead of relying on the context to infer their meanings “on the fly”, thereby improving reading speed and comprehension accuracy. This vocabulary “breadth” factor is traditionally considered to be the main driving force of the relationship between vocabulary knowledge and language processing. However, some researchers have recently proposed that vocabulary “depth”, which refers to the richness of word meanings, is also important (e.g., Binder et al., 2017). One way to measure vocabulary depth is to present participants with some root words and ask them to produce as many derivatives as possible for each word. As such, it is similar to a test of morphological processing. While the present vocabulary test was originally designed to be a breadth test, some may argue that it also taps into the depth component because of the morpho-syllabic nature of the Chinese script. Specifically, given that each Chinese character corresponds to a morpheme, it may be possible to infer a word’s meaning based on its constituent morphemes. Fortunately, some of the items used are idioms, whose meanings cannot be constructed directly from the characters. Nevertheless, in future vocabulary test development, it will be beneficial to take note on the need to separate the breadth and depth dimensions of vocabulary knowledge.

In his tutorial on test construction, Brysbaert (2024) highlighted the importance of making the tests readily and preferably freely available to the research community. Following such advice, we have uploaded the tests and the data reported in study to https://osf.io/v26nk/. Interested researchers can download the tests with no cost. The availability of normative performance from over 500 university students (Fig. 2) can help define the inclusion criteria in selecting appropriate participants in future studies. For example, one can specifically recruit participants who score below 66 and above 85 in the dictation test, which corresponds to the 25 th and 75 th percentiles in the norm, in a study that compares the reading performance between readers of low and high lexical expertise. Alternatively, researchers who adopted a between-subject design (e.g., in comparing young and old readers) can also use the tests to control for lexical expertise across groups. Furthermore, given that the tests (especially the vocabulary knowledge test) can be completed in a short time, it is also a nice idea to include these tests as part of the standard protocol in data collection and reporting. If two similar studies produce inconsistent results, and the participants in these studies differ in the dictation and vocabulary scores. Then, a reasonable next step is to examine whether and how individual differences in lexical expertise may have affected the results. More generally, having information about individuals’ language proficiency level can facilitate cross-study comparisons (e.g., in meta-analyses). As stated in Olson (2024), “inclusion of some form of proficiency assessment, even if not particularly relevant for the study’s research questions, will allow future researchers to effectively reference study results” (p. 180).

To use the dictation and vocabulary tests properly, several limitations of the tests should be noted. Firstly, these tests were developed for users of both simplified and traditional Chinese scripts. While this can be advantageous to those interested in testing users of both scripts, one can easily imagine that choosing items usable in both scripts is a difficult task, and tests designed specifically for one script may have more promising psychometric properties.

Secondly, both the dictation and the vocabulary tests include low-frequency words to increase the test difficulty so that they will be suitable for university students. The dictation test has only been validated with university students, who are typically proficient language users. It remains to be seen whether it is equally applicable to less proficient Chinese users in the public. On the other hand, the additional dataset shows that the vocabulary test continues to have reasonable performance in a community sample. Therefore, the low-frequency items may still be useful even for participants with diverse language proficiency levels. Indeed, Brysbaert et al. (2019) showed that words with low frequency in frequency corpus may be known by many language users (i.e., high word prevalence). Currently, word prevalence norm is not available in Chinese. When such data become available in the future, it will be beneficial to consider this factor when choosing items of appropriate difficulty level when constructing tests for Chinese proficiency. It is also possible to achieve a broader coverage of proficiency level when multiple tests are used in conjunction. For instance, when Wen et al. (2024) published LexCHI, a Chinese version of the LexTALE test, they validated it with L2 Chinese users. The test is thus potentially usable by less proficient Chinese readers. In contrast, native Chinese participants had near-ceiling performance in LexCHI (91–93 out of 100).

Thirdly, data collection was done only in southern China (Guangzhou and Hong Kong), although the Guangzhou group has participants from across mainland China. While the standard written Chinese is mostly the same across different geographical regions, the psychometric properties of the tests remain to be tested for Chinese users in, say, Taiwan and Singapore.

Finally, it is noteworthy that language processing is a complex activity that involves multiple dimensions of individual differences. While the dictation and vocabulary tests reported will be useful in measuring lexical expertise, they do not necessarily reflect other aspects of individual differences, such as syntactic awareness or the ability to make inferences based on the given text. To better understand individual differences, it will be important to develop relevant tests with good psychometric quality and to make them readily accessible to the research community.

## Supplementary Information

Below is the link to the electronic supplementary material.

Supplementary file1 (DOCX 38 KB)

## Data Availability

All data and materials are available at https://osf.io/v26nk/.

## Footnotes

1 With four choices, the chance level of answering a question correctly is 25%. With 15 pilot participants, 3.75 would be able to give correct answer by chance. The lower cutoff was set more lenient (keeping an item when at least three pilot participants answered correctly, which equaled to 20% of 15) to keep difficult items. The upper cutoff 80% was then decided based on the 20% difference from 100% correct.

2 The rationale for using this partial scoring method is to take into account the fact that each Chinese character is an independent unit that can plays a role in word recognition (Tsang et al., 2018). Thus, writing down a correct character correctly is considered to reflect partial lexical knowledge, even when other characters in the word are wrong. An alternative is to score correct–incorrect in a binary fashion based on the whole words. When this binary scoring approach was adopted, the average dictation performance declines (49.62% with binary scoring vs. 75.23% with partial scoring). However, the two scoring methods were themselves highly correlated (Pearson’s *r* = 0.931). Most importantly, the psychometric properties of the test remain similar. That is, a single factor model is obtained with EFA. The reliability is also satisfactory and of similar values (McDonald’s ω = 0.773; Cronbach’s α = 0.764). The supplementary analyses based on binary scoring can be found at https://osf.io/v26nk/.

3 The modified LexCHI was developed following the procedure of LexCHI (Wen et al., 2024). The primary purpose was to make the test more difficult because the original LexCHI was intended for those who learn Chinese as a second language. The modified LexCHI test and the preprint describing its development and use can be found at https://osf.io/3pvkr/.

## Code availability

The code used for analyses is available at https://osf.io/v26nk/.
