# Supplementary material for: Dictation and vocabulary knowledge tests for adult native Chinese readers
Source: Behav Res Methods. 2025 Apr 22;57(5):151. doi: 10.3758/s13428-025-02669-4 (PMC12014802; doi:10.3758/s13428-025-02669-4)
Supplement: Supplementary file 1 — Supplementary file1 (DOCX 38 KB) [file 13428_2025_2669_MOESM1_ESM.docx]

Supplementary analysis of dictation score

In the original analyses, the dictation score was coded based on the number of characters correctly produced, instead of making a binary correct-incorrect scoring based on the entire words. The rationale for doing so is to account for the compositional nature of Chinese words, such that each correct character is considered as reflecting partial knowledge to the whole-word. To examine how this partial scoring approach may deviate from the binary scoring approach, a series of supplementary analyses was conducted with Group 1 participants.

When each dictation response was coded as correct or incorrect, the exploratory factor analysis (EFA) with a tetrachoric correlation matrix again shows that a one-factor model can adequately capture the data (scree plot in Figure S1). Moreover, none of the item has a factor loading or item-rest correlation below 0.1 (Table S1). In other words, contrary to the original EFA that results in discarding one item (d11), the binary scoring approach allows retaining all items.

Figure S1. Scree plot for supplementary analysis of the dictation test.


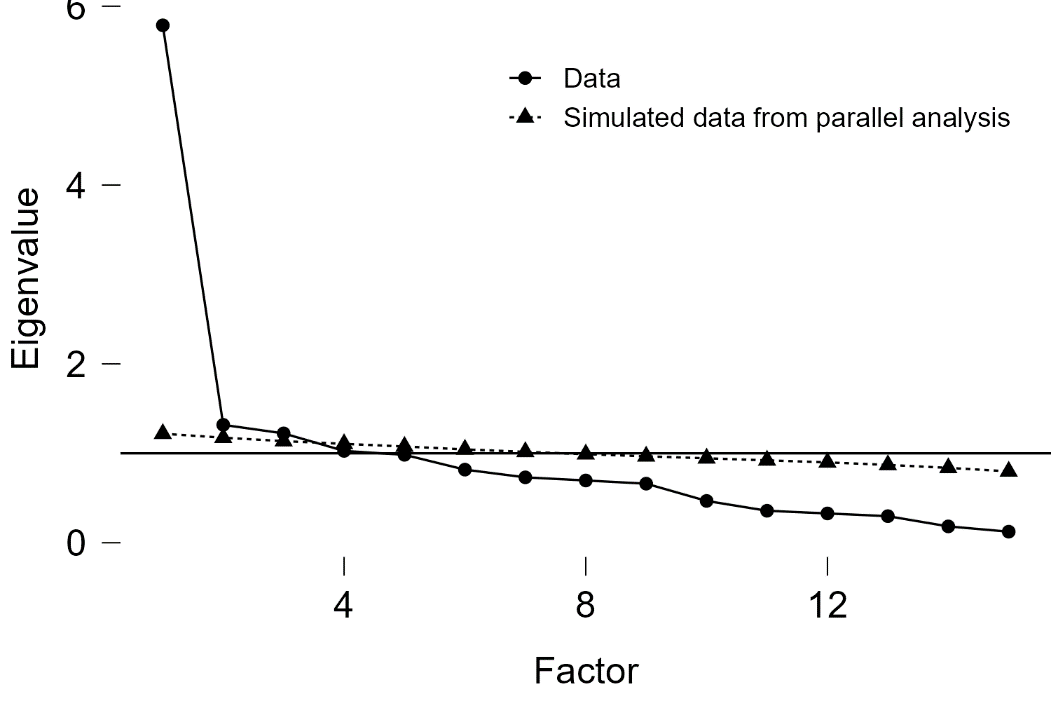


Table S1. Factor loadings and item-rest correlation of items in dictation test in Group 1 using an alternative binary scoring approach (correct-incorrect).

| Item_id | Factor loading | Item-rest correlation |
| --- | --- | --- |
| ad06 | 0.834 | 0.578 |
| ad05 | 0.809 | 0.546 |
| ad08 | 0.708 | 0.486 |
| ad13 | 0.698 | 0.453 |
| ad07 | 0.673 | 0.45 |
| ad15 | 0.666 | 0.372 |
| ad14 | 0.611 | 0.39 |
| ad03 | 0.562 | 0.302 |
| ad10 | 0.535 | 0.374 |
| ad09 | 0.513 | 0.36 |
| ad12 | 0.507 | 0.345 |
| ad04 | 0.459 | 0.275 |
| ad02 | 0.374 | 0.178 |
| ad11 | 0.318 | 0.216 |
| ad01 | 0.21 | 0.146 |

When all items were included under a single factor, the internal reliability of the dictation score was similar to the original partial scoring approach. Specifically, the Cronbach's α and McDonald's ω were 0.764 and 0.773, respectively (as compared to 0.761 and 0.771 with partial scoring).

As expected, participants’ overall performance in the dictation test dropped with the binary scoring approach (mean = 49.62%, as compared to 75.23% with partial scoring). However, the two scoring methods were themselves highly correlated (Pearson’s *r* = 0.931). The overall pattern of correlation with other measures was also highly similar between the two scoring methods (Table S2).

Table S2. Correlations with other measures

|  | Dictation score  (partial scoring) | Dictation score  (binary scoring) |
| --- | --- | --- |
| Number of hours of Chinese reading per week | 0.21 | 0.17 |
| Self-rated Chinese proficiency | 0.18 | 0.22 |
| Chinese subject score in Gaokao | 0.10 | 0.14 |
| Chinese subject grade in HKDSE | 0.36 | 0.34 |
| Lexical decision standardized reaction time | -0.21 | -0.19 |
| Lexical decision error rate | -0.54 | -0.48 |

Based on these analyses, it can be concluded that while the binary scoring method will keep all items, the overall psychometric properties of the dictation test are largely independent of the way the dictation score was computed.
